# Feasibility and mechanism analysis of Reduning in the prevention of sepsis-induced pulmonary fibrosis

**DOI:** 10.3389/fphar.2022.1079511

**Published:** 2022-12-20

**Authors:** Ziyi Wang, Yuxin Liu, Feng Chen, Haiyan Liao, Xuesong Wang, Zhe Guo, Zhong Wang

**Affiliations:** ^1^ School of Clinical Medicine, Tsinghua University, Beijing, China; ^2^ Department of Cardiovascular Thoracic Surgery, Tianjin Medical University General Hospital, Tianjin, China

**Keywords:** sepsis, Reduning, ERBB2, pulmonary fibrosis, MAPK

## Abstract

**Introduction:** The increasing mortality in patients with sepsis-induced pulmonary fibrosis owes to a lack of effective treatment options. This study aims to explore the possibility and possible targets of Reduning in the prevention of sepsis-related pulmonary fibrosis.

**Methods**: The active components and targets of Reduning were searched and screened from the database and analysis platform of traditional Chinese medicine (TCM) system pharmacology. GeneCards, human genome database, DisGeNET database, and the OMIM database were checked to determine the targets associated with sepsis-induced pulmonary fibrosis. DAVID Bioinformatics Resources 6.8 was used for GO and KEGG enrichment analysis to predict its possible signaling pathways and explore its molecular mechanism. The protein–protein interaction (PPI) network was used to identify key active components and core targets. Molecular docking technology was applied to screen the complexes with stable binding of key active components and core targets. Molecular dynamics simulations were used to verify the binding stability and molecular dynamics characteristics of the complexes. The protective effect of RDN on sepsis-induced pulmonary fibrosis was verified by *in vitro* and *in vivo* experiments.

**Results:** There were 319 shared targets between sepsis-induced pulmonary fibrosis and RDN. GO enrichment analysis showed that they mainly regulated and participated in the positive regulation of kinase activity, mitogen-activated protein kinase (MAPK) cascade, and protein phosphorylation. KEGG enrichment analysis showed that they were mainly enriched in the mitogen-activated protein kinase cascade signaling pathway, the calcium signaling pathway, the apoptosis pathway, and other signaling pathways. The results of molecular docking and molecular dynamics simulations showed that the active components, stigmasterol, beta-sitosterol, and quercetin, had good binding activities with ERBB2, and they exhibited good stability. Molecular validation experiments confirmed RDN could alleviate lung fibrosis induced by cecum ligation and puncture (CLP), in parallel with the inhibition of the ERBB2-p38 MAPK pathway in mouse alveolar macrophages (AMs).

**Discussion:** Reduning may prevent sepsis-induced pulmonary fibrosis by regulating the ERBB2-p38 MAPK signaling pathway, which provides a possibility for the prevention of sepsis-induced pulmonary fibrosis with traditional Chinese medicine.

## 1 Introduction

Sepsis-induced acute respiratory distress syndrome (ARDS), a syndrome characterized by acute respiratory failure and intractable hypoxemia, may trigger persistent fibrosis (Meyer et al., 2021). Patients with sepsis-induced pulmonary fibrosis have characteristics such as reduced pulmonary ventilation, diffusion function, and changes in metabolic function, which further aggravate the mortality of ARDS (Luyt et al., 2020). Since the formation of pulmonary fibrosis is hard to reverse, it is evidently important to find effective drugs to prevent sepsis from progressing to pulmonary fibrosis (Henderson et al., 2020).

In recent years, scholars have gradually deepened their exploration of the possible effects and related mechanisms of TCM in the prevention and treatment of acute and chronic lung injury caused by sepsis (Chen et al., 2019; Yin et al., 2021). Especially since the outbreak of coronavirus disease 2019 (COVID-19), the use of traditional Chinese medicine (TCM) has achieved outstanding effects in improving lung function, reducing mortality, and improving patient prognosis (Liang et al., 2020; An et al., 2021). Reduning is a TCM injection composed of Artemisia annulosa, honeysuckle, and gardenia, which is recommended for the treatment of critical COVID-19 patients by “the Diagnosis and Treatment Protocol for COVID-19 (Trial ninth Edition).” Our previous studies confirmed that Reduning could improve sepsis-induced acute lung injury (SALI) by downregulating LPS-induced apoptosis of human umbilical vein endothelial cells (HUVECs) through the PI3K–AKT pathway and thereby improving vascular endothelial barrier function (Wang et al., 2022a). Existing studies have shown that, the basic treatment of Western medicine, supplemented with Reduning, could significantly improve the clinical symptoms, pulmonary function, and prognosis of patients with idiopathic fibrosis (Wang et al., 2020; Shi et al., 2022). Animal experiments also confirmed that Reduning has a certain protective effect on bleomycin-induced pulmonary fibrosis in rats (Zhang et al., 2018). However, the mechanisms of its effect on inflammatory lung injury have not been explored.

The rise of network pharmacology has provided ideas for revealing the mechanism of TCM compounds in the prevention and treatment of diseases recently. Network pharmacology integrates the advantages of pharmacology, proteomics, and systems biology and has the characteristics of holistic and systematic research. These advantages and characteristics are consistent with the holistic view of TCM, which is conducive to reveal the scientific connotation of multi-component, multi-target, multi-pathway, and holistic regulation of diseases in TCM (Shi et al., 2022). This study attempted to analyze the possibility and possible molecular mechanism of Reduning in the prevention of sepsis-induced pulmonary fibrosis from the perspective of network pharmacology in order to provide scientific basis for the prevention and further research of sepsis-induced pulmonary fibrosis.

## 2 Materials and methods

### 2.1 Bioinformatics methods

#### 2.1.1 Component target information query

The analysis platform and database system pharmacology of traditional Chinese medicine (TCMSP, https://tcmspw.com/tcmsp.php/) on artemisinin, honeysuckle, and gardenia correspond to active component retrieval. Taking oral bioavailability (OB) ≥ 30% and drug like (DL) ≥ 0.18 as screening conditions, 49 active compounds of TCM were screened. The PubChem database (https://pubchem.ncbi.nlm.nih.gov/) was used to retrieve 49 active compounds, which was be the primer number input to predict the 782 targets using the SwissTargetPrediction platform.

#### 2.1.2 Search for disease target

GeneCards, human genome database (https://www.disgenet.org/), the NCBI database (https://www.ncbi.nlm.nih.gov/), and the OMIM database (https://omim.org/) were used to obtain the target genes of disease. The target retrieval was performed using “pulmonary fibrosis in sepsis” as the search term, and the species was set as human. The disease targets retrieved from the database were merged and de-weighted to obtain 2,121 final disease targets.

#### 2.1.3 Venn diagram

**FIGURE 1:**
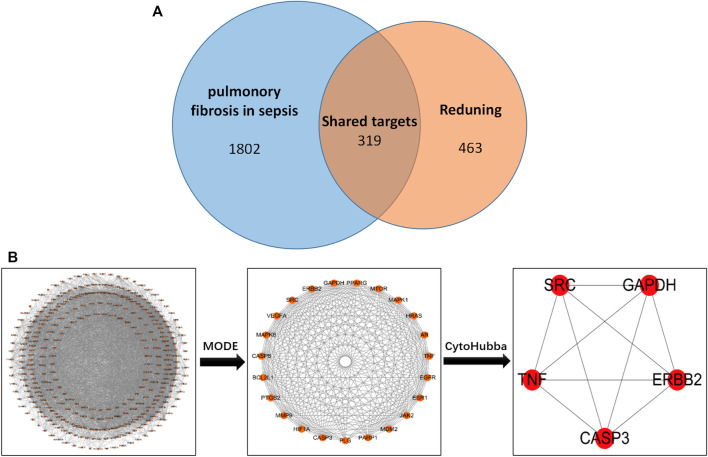
**(A)** Drug targets for RDN and sepsis-induced pulmonary fibrosis. **(B)** PPI network of 319 overlapping gene symbols.

The screened drug targets and disease targets were input into Venn diagram construction software, Venny 2.1, and 319 common targets were obtained, which were used as predicted targets of drugs acting on diseases for subsequent analysis, as shown in Figure 1.

#### 2.1.4 PPI network construction and key target screening

The common targets of drugs and diseases were input into the STRING database (https://string-db.org/cgi/input.pl) to construct the PPI network, and the biological species was set as “*Homo sapiens*” to obtain the PPI network. There are 319 nodes and 5,487 edges in the network, with an average degree of 34.4 (Figure 1B is the PPI network diagram exported from the STRING database). TSV files obtained from the STRING database were imported into Cytoscape 3.7.2 software, and the PPI network was analyzed by the MODE module and cytoHubba to screen out the core target genes (Table 1).

**TABLE 1:** Shared key targets of sepsis and RDN.

Target	Degree value
SRC	140
TNF	192
CASP3	126
GAPDH	167
ERBB2	107

#### 2.1.5 GO and KEGG enrichment analysis

Shared targets of RDN and sepsis-induced pulmonary fibrosis were imported into DAVID Bioinformatics Resources 6.8 (https://david.ncifcrf.gov/home.jsp) to analyze GO enrichment, including biological processes (in the process, BP), cell component (CC) enrichment, and molecular function (MF). *p*-value cutoff = 0.05, q-value cutoff = 0.05, and the rest defaulted to original settings. The shared targets were imported into DAVID Bioinformatics Resources 6.8 to clarify KEGG pathway enrichment. We chose the top 10 pathways according to *p*-value and used microbioinformatics (http://www.bioinformatics.com.cn/) to visualize.

#### 2.1.6 Molecular docking analysis

The compound name, molecular weight, and 3D structure of the active compounds were determined using the PubChem database, and the corresponding 3D structures of the active compounds were downloaded from the RCSB PDB database (http://www.rcsb.org/). Then, AutoDock software was used to prepare the ligands and proteins required for molecular docking. The crystal structure of the target protein was created by removing water molecules, hydrogenation, modified amino acids, optimized energy and adjusted field parameters, and then the low-energy conformation of the ligand structure was satisfied.

#### 2.1.7 Molecular dynamic simulation (MD)

GROMACS 2019.6 was selected as dynamics simulation software, the docking result was taken as the initial conformation, amber14sb was used as the protein force field, and GAFF2 (generation Amber force field) was used for small molecules. The TIP3P water model was used to add the TIP3P water model to the complex system to establish the water box, and sodium ions were added to construct the equilibrium system (Van Der Spoel et al., 2005). In the elastic simulation, the electrostatic interactions were handled by the Verlet and CG algorithms and the particle mesh Ewald (PME) method, respectively, and the steepest descent method was used to minimize the energy for a maximum number of steps (50,000). The cutoff distance of Coulomb force and van der Waals force radii was set to 1.4 nm, and the MD simulation was carried out for 50 ns at normal temperature and pressure using the equilibrium system of regular system (NVT) and the isothermal isobaric system (NPT). In the MD simulation process, the hydrogen bonds involved were constrained by the LINCS algorithm and calculated by the PME method. The integration step was set at 2 fs and the cutoff value was set as 1.2 nm. The non-bonded interaction cutoff was set to 10 A. The simulated temperature was controlled to 300 K using the V-rescale temperature coupling method, and the pressure was controlled to 1 bar using the Berendsen method. Root mean square deviation (RMSD) was applied to observe the allostery of local sites during the simulation (the fluctuation cutoff point was set to 0.2). The radius of gyration (Rg) evaluated the closeness of the architecture. In the simulation process, root mean square function (RMSF) was used to observe the allosteric situation of local sites. Solvent accessible surface area (SASA) was used to observe the solvent accessible surface area during the simulation. HBNUM exhibited hydrogen bonding between protein ligands during the simulation.

### 2.2 Regents

Anti-p-p38 MAPK (Thr180/Tyr182) antibody, anti-p38 MAPK antibody, and FITC-anti-F4/80 antibody were from Cell Signaling Technology. Anti-p-ERBB2/HER2 (phospho Y877) antibody, anti-ERBB2/HER2 antibody, anti-GAPDH antibody, anti-CD206 antibody, and Alexa Fluor 647-anti-ERBB2 (phospho Y877) antibody were from Abcam. A macrophage isolation kit (peritoneum) was purchased from Miltenyi Biotec.

### 2.3 Animal model

Male wild-type (WT) C57BL/6J mice aged 6–8 weeks were purchased from Beijing Huafukang Biotechnology Co., Ltd. All mice were subsequently bred and settled under specific pathogen-free (SPF) conditions at Tsinghua University. In our study, we anesthetized WT mice with an intraperitoneal injection of tribromoethanol (10 mg/kg). After midline laparotomy, for cecum ligation and puncture (CLP) groups, the cecum was exposed and ligated immediately below the ileocecal valve, causing intestinal obstruction. After two punctures with an 18G needle, the cecum was placed back and the abdominal wall was sealed. Reduning group and CLP+Reduning group were administered with Reduning (50 or 100 mg/kg) by intraperitoneal injection once per day for 7 days before the operation. For the sham group, the cecum was exposed but did nothing, and then the abdominal wall was closed. BAL fluid (BALF) was collected by rinsing the alveolar cavity with 1 ml precooled PBS, and it was stored at −20°C. The right lungs were embedded in 10% formalin prepared for morphology examination. For all these groups, normal saline was injected intraperitoneally just after the operation to simulate clinical fluid replenishment. At 21 days after the operation, mice were sacrificed, and the inferior lobe of the right lung was used as the study sample. All study designs were supervised and approved by the Ethics Committee of Beijing Tsinghua Changgung Hospital (protocol code NCT05095324).

### 2.4 Masson staining

After fixation in 4% paraformaldehyde, the lung tissues were dehydrated, embedded in paraffin, and sliced for Masson staining. The morphological structure, inflammatory infiltration, and degree of fibrosis of the lung tissues were evaluated under a microscope after the sections were stained. The Ashcroft score for pulmonary fibrosis was performed on the lung histopathological sections of each mouse, and the scoring criteria were as follows (Carl-Mcgrath et al., 2008): 0: normal lung; 1–2: slight thickening of alveoli and bronchial walls; 3–4: moderate thickening of alveolar and bronchial walls, but no evident destruction of lung structure; 5–6: more pulmonary fibrosis with specific lung structure destruction, fiber bundle formation, and small fiber aggregation; 7: severe pulmonary structure distortion, extensive fibrosis, and honeycomb lung; and 8: pulmonary fibrosis.

### 2.5 Immunohistochemistry (IHC)

IHC was performed following the manufacturers’ protocols. Sections were backstained, dehydrated, removed, and mounted with modified methylethoxylin. All regiments included appropriate positive and negative controls. The percentage of F4/80 protein positive in the lung tissue was derived from three separate data.

### 2.6 Immunofluorescence (IF)

After the sections were dewaxed and hydrated, lx EDTA antigen repair solution (PH 9.0) was used for antigen repair. After PBS cleaning, endogenous peroxidase block-agent was added to the tissue sections to block the activity of endogenous peroxidase. Goat serum was added to the tissue sections for sealing and incubated in a wet box at room temperature for 30 min. After that, primary antibody diluted with sterile PBS (a-SMA antibody concentration 1:500) was added to the covered tissue sections and incubated at 4°C overnight. An appropriate amount of immunofluorescent secondary antibody was added and was inoculated for 1 h against light at room temperature. Finally, the nucleus was stained with DAPI dye solution, and the tablet was sealed with an anti-fluorescent quench agent.

### 2.7 Macrophage isolation experiment

Cells from mice BALF were collected and harvested after centrifugation at 1,000 rpm for 10 min at 4°C and suspended in precooled sterile PBS containing 2% FBS. Mouse alveolar macrophages (AMs) were isolated using a macrophage isolation kit (peritoneum) following the manufacturer’s instructions. Then, Western blot was applied to detect the expression level of the indicated proteins.

### 2.8 Cell culture

AMs were cultured with Dulbecco’s modified Eagle medium (DMEM) and high glucose (Gibco, United States) under standard conditions (37°C, 5% CO_2_) in the laboratory of Tsinghua Changgung Hospital. The experiments were exhibited after two passages. Cells were incubated with LPS (100 ng/ml). After the indicated length of time, cell culture supernatant and cells were collected. The supernatants were analyzed by enzyme-linked immunosorbent assay (ELISA) to evaluate pro-inflammatory and anti-inflammatory factors. Then, Western blot were applied to detect the expression level of the indicated proteins.

### 2.9 Western blot

ERBB2 proteins were evaluated by Western blotting. Total protein was extracted according to the standard protein extraction protocol. The protein concentration was quantified by a BCA protein assay kit. The sample was electrophoretic in a 10% SDS-PAGE gel and transferred to a polyvinylidene fluoride film. The film is then sealed overnight with 5% milk at 4°C. The membrane was rinsed three times with Tris-buffered saline Tween-20 (TBST) and incubated with secondary antibodies at room temperature for 40 min. After washing with TBST again, the protein signal was observed, and the membrane signal was displayed by an enhanced chemiluminescence Western blot detection system. The staining intensity of the strip was measured using ImageJ software. The antibodies used in our study are described in the previous “reagents” section. Protein expression levels were defined as gray values normalized to the steward gene GAPDH and expressed as multiples of the control. All experiments were performed three times and three times independently.

### 2.10 Data processing and statistical analysis

Data were expressed as the mean ± SD. The differences between variables were compared using one-way ANOVA or a two-tailed Student’s t-test using SPSS software (version 22.0; United States). *p* < 0.05 was considered statistically significant. Moreover, Prism 8 (GraphPad Software, United States) was responsible for visualizing.

## 3 Results

### 3.1 Construction of Reduning—compound—target network

**FIGURE 2:**
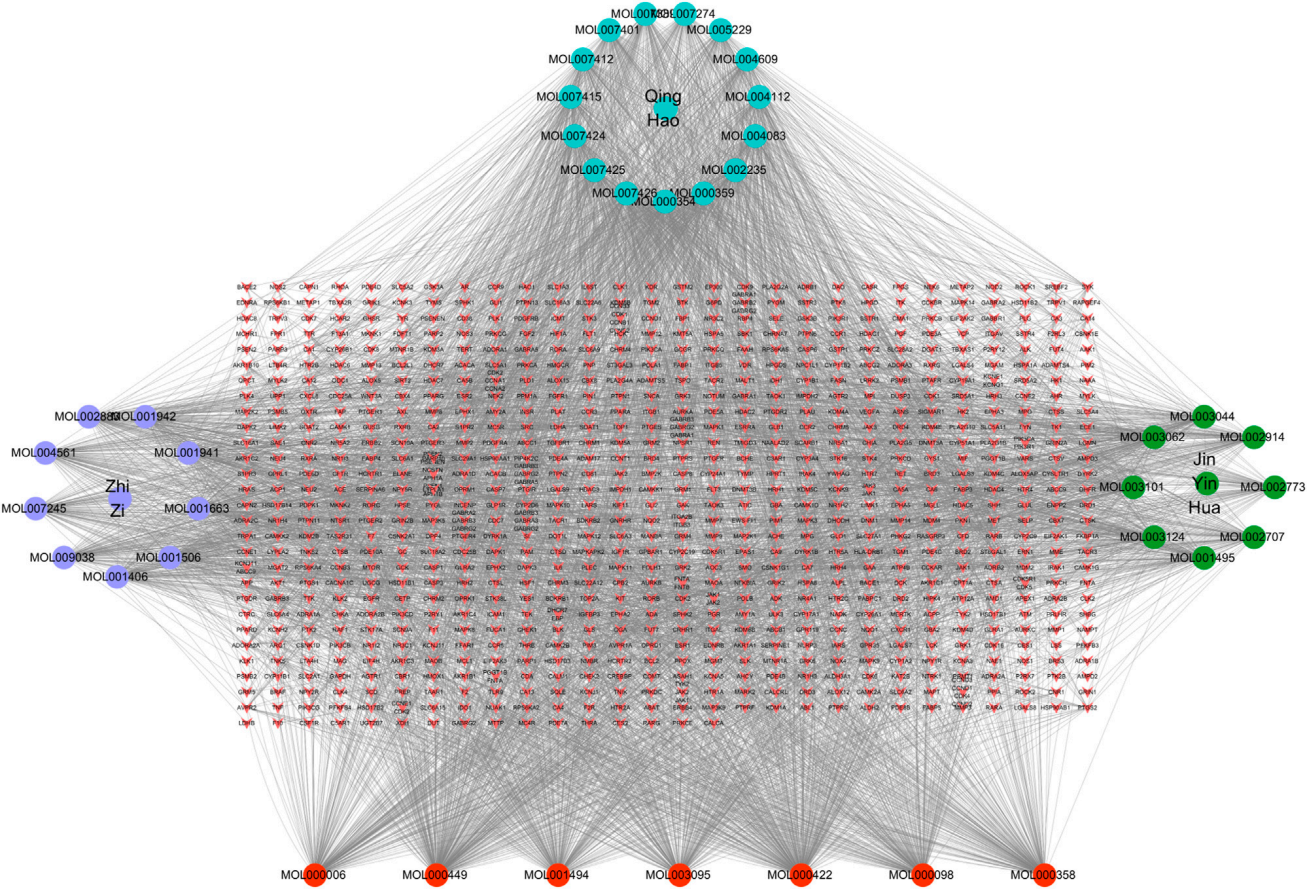
TCM–component–target–disease network. In the network, the purple, green, and cyan circles are the TCM and the three corresponding herbs, and the red circles are the compounds common to the three herbs. The targets corresponding to the active ingredients form the red inverted triangle.

In order to better understand the intricate relationship among Reduning, active components, and corresponding targets, a network diagram of TCM, compound, and target was constructed based on the included TCM, active components, and target sites. The network was imported into Cytoscape 3.7.2 software to draw the network diagram, and the results are shown in Figure 2. Topological analysis selected the top five compounds by degree as key compounds, as shown in Table 2.

**TABLE 2:** Key active compounds.

Compound	Degree value
Quercetin	504
Kaempferol	402
Beta-sitosterol	279
Stigmasterol	227
Mandenol	202

### 3.2 GO and KEGG enrichment analysis of shared targets

**FIGURE 3:**
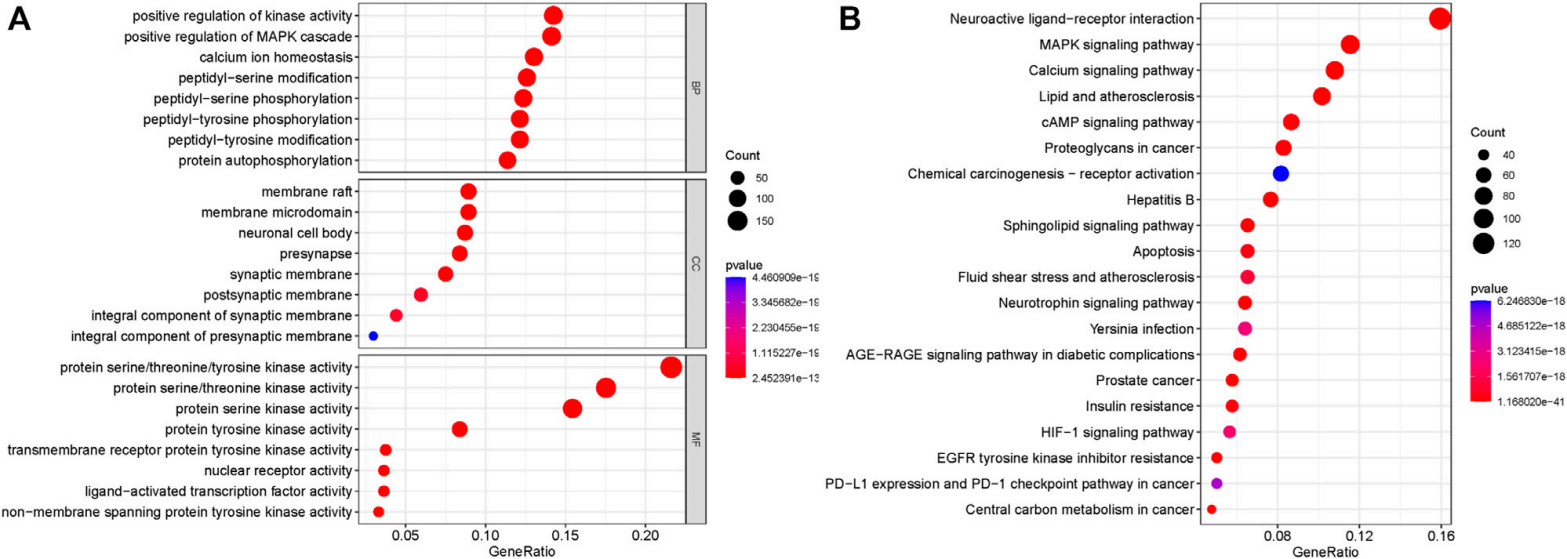
GO analysis and KEGG pathway enrichment analysis of the 319 shared targets. **(A)** GO analysis of the shared targets. The abscissa represents the number of targets; the left represents BP, CC, and MF; and the color represents the *p*-value. The smaller the *p*-value, the more red the color is, while the larger the *p*-value, the more blue the color is. **(B)** KEGG pathway enrichment analysis of the shared targets. The abscissa represents the number of genes enriched, the left represents the pathway name, and the color represents the *p*-value. The smaller the *p*-value, the more red the color, and the larger the *p*-value, the more blue the color.

In order to further explore which pathways are mainly affected by the shared targets of diseases and TCM, we conducted GO and KEGG enrichment analysis on the shared targets. A total of 1,634 GO entries were enriched. There were 217 cell components (CCs), which mainly located membrane rafts, membrane microdomains, neuronal cell bodies, synaptic membranes, postsynaptic membranes, and components of presynaptic membranes. There were 3,477 biological processes (BP), which mainly regulated and participated in the processes of positive regulation of kinase activity, positive regulation of MAPK cascade, calcium homeostasis, peptidyl-serine modification, peptidyl-serine phosphorylation, peptidyl-tyrosine phosphorylation, peptidyl-tyrosine modification, and protein self-phosphorylation. There were 426 molecular functions (MFs), mainly manifested in protein serine/threonine/tyrosine kinase activity, transmembrane receptor protein tyrosine kinase activity, nuclear receptor active ligand-activated transcription factor activity, and non-transmembrane protein tyrosine kinase activity. The top eight enrichment items with *p*-value were selected, and according to the *p*-value, Q-value of each item, and the number of genes enriched on it, the ggplot2 package of the R language was used to draw the bubble map of GO analysis. The abscissa represents the number of targets, the left represents BP, CC, and MF, and the color represents the *p*-value. The smaller the *p*-value, the more red the color is; the larger the *p*-value, the more blue the target is, as depicted in Figure 3A.

A total of 191 KEGG pathways were enriched. The top 20 enrichment items with *p*-value were selected, and according to the *p*-value, Q-value of each item, and the number of genes enriched on it, the ggplot2 package of the R language was used for visual analysis of the results. The abscissa represents the number of genes enriched, the left represents the pathway name, and the color represents the *p*-value. The smaller the *p*-value, the more red the color, and the larger the *p*-value, the more blue the color. As shown in Figure 3B, shared targets were mainly enriched in the MAPK signaling pathway, the calcium signaling pathway, the AMP signaling pathway, the sphingolipid signaling pathway, the apoptosis pathway, the neurotrophic factor signaling pathway, the AGE-RAGE signaling pathway, the HIF-1 signaling pathway, and the PD-1 checkpoint in diabetic complications.

### 3.3 The top five complexes of key active compounds and targets were selected and analyzed by molecular docking analysis

**FIGURE 4:**
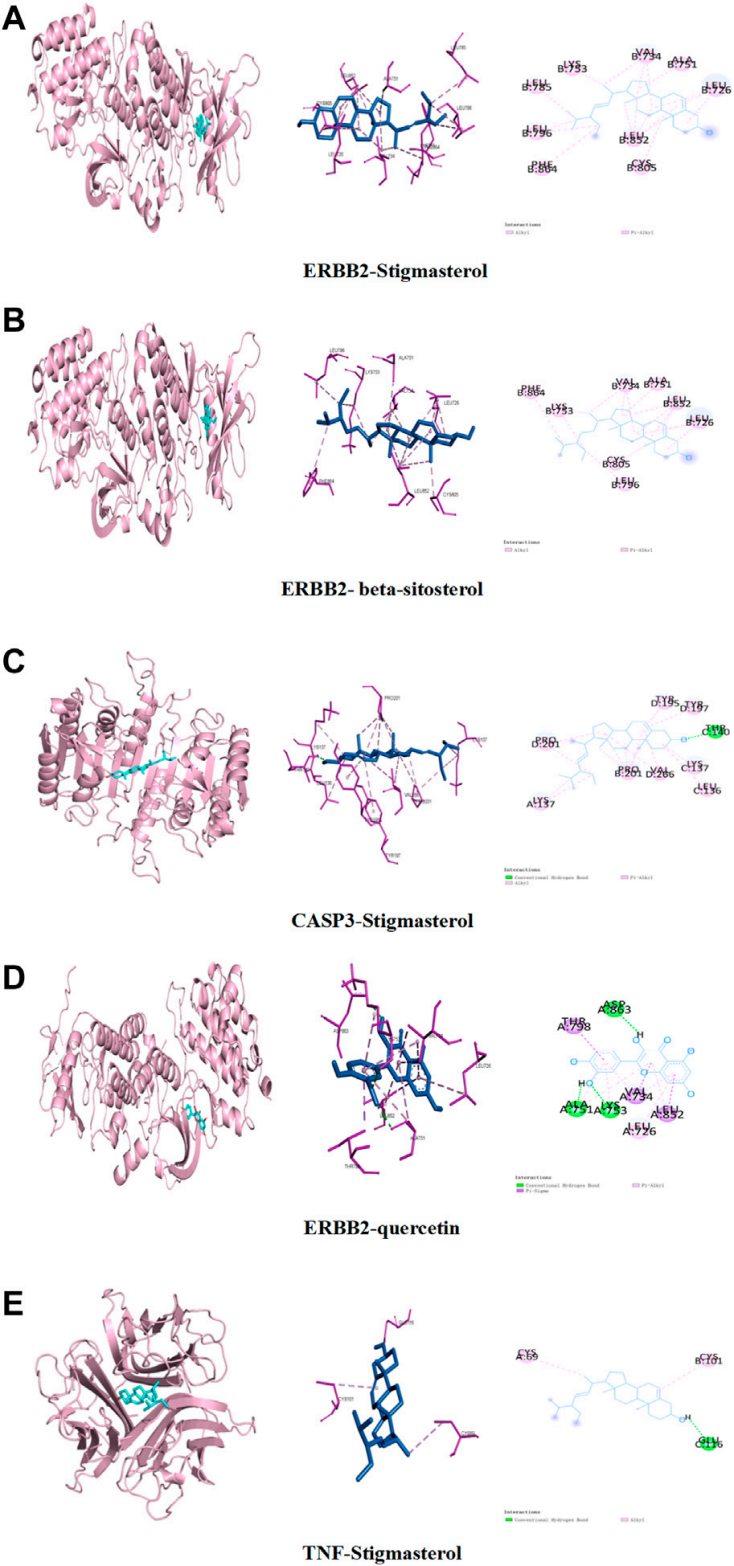
Binding studies of selected ingredient–target interactions. **(A)** ERBB2 with stigmasterol; **(B)** ERBB2 with beta-sitosterol; **(C)** caspase-3 with stigmasterol; **(D)** TNF with stigmasterol; **(E)** ERBB2 with quercetin.

In order to clarify the interaction between active components and targets and to predict the binding mode and affinity of complexes, AutoDock software was used to dock the aforementioned five key components and five targets. The compound names, molecular weights, and 3D structures of the active ingredients were determined from the PubChem database, and then, the corresponding 3D structures of the active ingredients were downloaded from the RCSB PDB database (http://www.rcsb.org/). AutoDock software was used to prepare the ligands and proteins required for molecular docking, remove water molecules, hydrogenate, modify amino acids, optimize energy, adjust force field parameters for the crystal structure of the target protein, and then meet the low-energy conformation of the ligand structure. Finally, the five key target structures and five key active components were used for molecular docking, and the affinity (kcal/mol) value represents the binding ability of the two. Finally, Discovery Studio and PyMol software were used to analyze and observe the docking results. The key active components stigmasterol, kaempferol, quercetin, beta-sitosterol, and corymbosin were used to perform molecular docking verification with the key targets SRC, TNF, CASP3, GAPDH, and ERBB2. The results showed that all 25 groups had good binding activity. The top five results were selected and analyzed. Table 3 and Figure 4 depict these relationships.

**TABLE 3:** Molecular docking binding energy.

Num	Compound	Target	Binding energy (kcal/mol)
1	Stigmasterol	ERBB2	−10.5
2	Beta-sitosterol	ERBB2	−10
3	Stigmasterol	CASP3	−9.6
4	Stigmasterol	TNF	−9.4
5	Quercetin	ERBB2	−9.4
6	Quercetin	GAPDH	−9.2
7	Beta-sitosterol	CASP3	−9.2
8	Stigmasterol	SRC	−9.1
9	Quercetin	TNF	−9.1
10	Kaempferol	GAPDH	−8.9
11	Corymbosin	GAPDH	−8.9
12	Kaempferol	TNF	−8.8
13	Kaempferol	ERBB2	−8.8
14	Beta-sitosterol	SRC	−8.8
15	Kaempferol	SRC	−8.7
16	Corymbosin	TNF	−8.5
17	Corymbosin	SRC	−8.3
18	Quercetin	SRC	−7.9
19	Quercetin	CASP3	−7.8
20	Beta-sitosterol	GAPDH	−7.7
21	Corymbosin	ERBB2	−7.6
22	Stigmasterol	GAPDH	−7.5
23	Kaempferol	CASP3	−7.5
24	Corymbosin	CASP3	−7.4
25	Beta-sitosterol	TNF	−6.3

### 3.4 The complexes of active compounds and ERBB2 were further analyzed by MD

**FIGURE 5:**
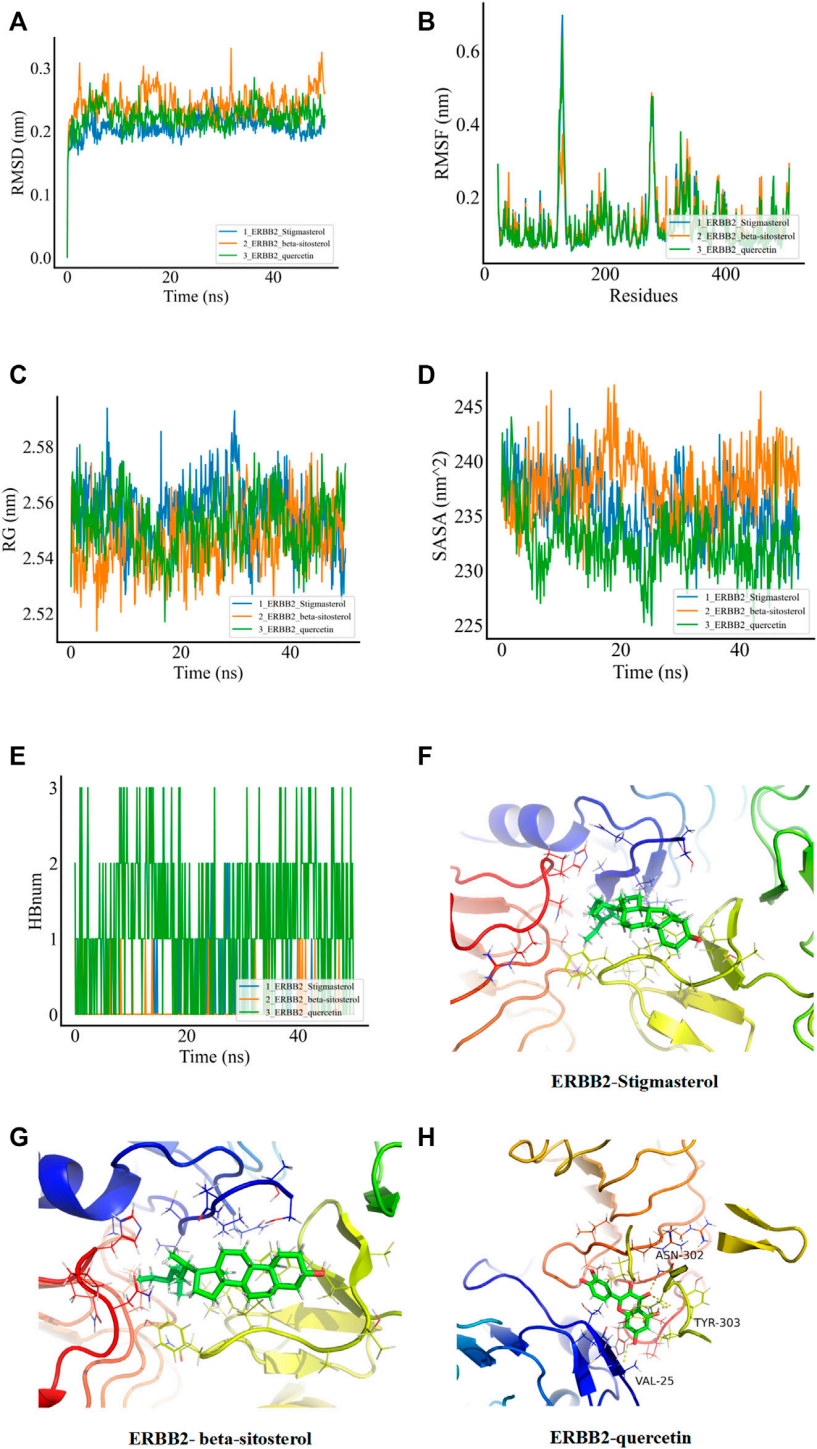
MDs of the ligand receptor complex. **(A)** Root mean square deviation (RMSD) of complex MDs. **(B)** RMSF changes in complex MDs. **(C)** Rg changes of complex MDs. **(D)** Solvent accessible surface area (SASA) of complex MDs. **(E)** Number of hydrogen bonds of complex MDs (HBNUM). **(F–H)** Molecular dynamics simulation of stable attitude and energy decomposition.

In order to verify whether the binding of the three groups of complexes with ERBB2 is stable, MD was applied to explore the stability and kinetic characteristics of the complexes in aqueous solution. The atomic root mean square deviation (RMSD) was used to measure the stability of the system, and the RMSD fluctuation range was small in the three groups of dynamics simulations, which could quickly stabilize to 0.2 nm after the beginning of the simulation (Figure 5A). RMSF can reflect the fluctuation in the simulation process, and the results show that the three groups of simulations share similar binding patterns, and their flexibility ranges are basically the same (Figure 5B). The radius of gyration (Rg) was used to evaluate the closeness of the architecture. The fluctuation amplitude simulated by the three groups was consistent with RMSD. As shown in Figure 5C, the stigmasterol complex with ERBB2 (group I) was slightly larger in magnitude than the beta-sitosterol complex with ERBB2 (group II) and the quercetin complex with ERBB2 (group III). SASA was used to explore the solvent-accessible surface of the protein surface, and it was found that the SASA of the protein in the range of 0–50 ns steadily decreased and the protein gradually contracted (Figure 5D). The number of HBNUM between protein ligands during simulation was further calculated. The first group was stable at one to two contacts, the second group was stable at 0–1 contacts, and the third group was fluctuating at one to three contacts (Figure 5E). Further analysis of the corresponding binding modes revealed that the first two stable binding postures were small molecules firmly adsorbed in the hydrophobic cavities around the B-sheet and A-helix (Figures 5F,H). Figure 5G illustrates the stable binding of small molecules to VAL-25, ASN-302, and TYR-303 of the ERBB2 (HER2) protein in the third group of the simulation system.

### 3.5 Reduning attenuates pulmonary fibrosis in the CLP-induced mice model

**FIGURE 6:**
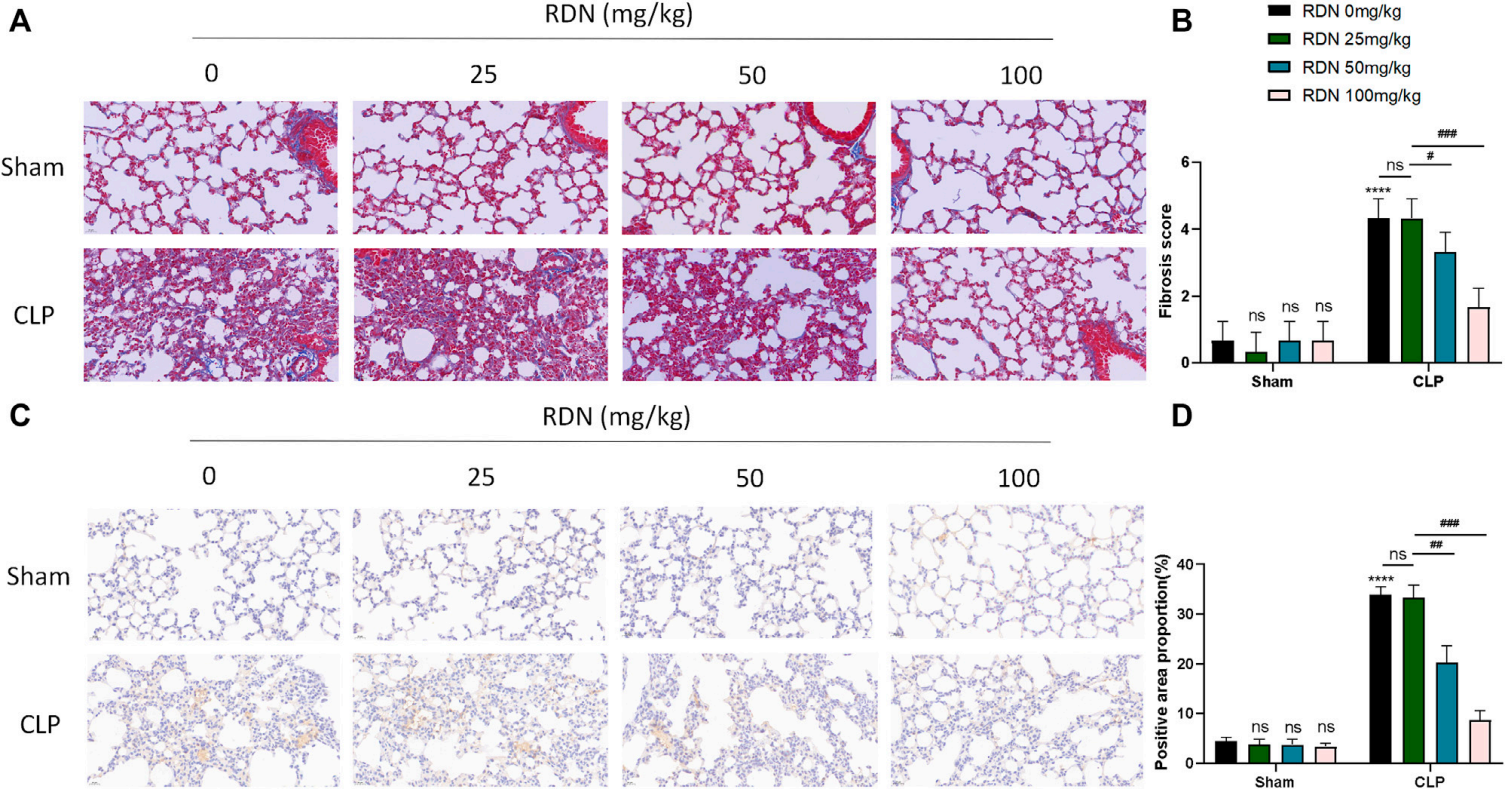
Expression level of fibrosis in mouse lung tissue 21 days after CLP operation. **(A,B)** Masson staining of mouse lung tissue. **(C,D)** IHC staining of mouse lung with α-SMA. Data are presented as mean ± SD (n = 3 per group) of the representative data from three independent experiments; ****p < 0.001, ###*p* < 0.005, ##*p* < 0.01, and #*p* < 0.05. The asterisk (*) represents statistically significant difference between the indicated group and the Con group.

As shown in Figures 6A,B, in the the CLP group, blue collagen fibers were deposited in the lung tissue, showing diffuse distribution, collapse of alveolar structure, evident thickening of the alveolar septum, and abnormal deposition of extracellular matrix. The CLP+RDN (50/100 mg/kg) group showed scattered collagen fiber deposition in the lung tissue, which was lighter than the CLP group. In the sham group, the CLP+RDN (25 mg/kg) group, and the RDN group, no notable collagen fiber deposition was observed in the lung tissue (*p* > 0.05). As shown in Figures 6C,D, the expression level of α-SMA protein in the lung tissue was observed by IHC. The expression of α-SMA in the lung tissue of the CLP group was significantly higher than that of the sham group. There was a small amount of α-SMA expression in the lung tissue of the CLP+RDN (50/100 mg/kg) group, which was lighter than the CLP group. In the sham group, there was no notable α-SMA expression in the lung tissues of the CLP+RDN (25 mg/kg) and the RDN groups, compared with the sham group (*p* > 0.05).

### 3.6 Reduning could downregulate p-ERBB2 located in the AMs in the CLP-induced pulmonary fibrosis model

**FIGURE 7:**
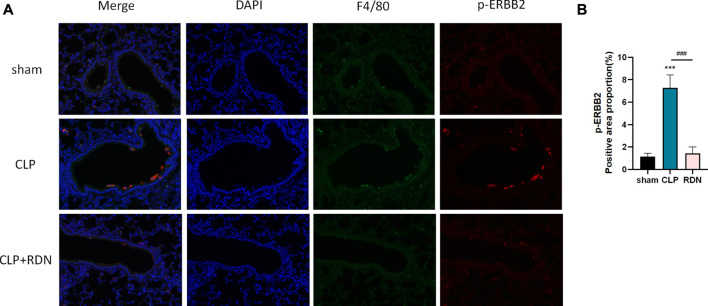
Interaction between ERBB2 activation and AMs; **(A)** IF was applied to detect the macrophage biomarker (F4/80) and the activation forum of ERBB2 (p-ERBB2). **(B)** Reduning could reduce the expression level of p-ERBB2 enhanced in the CLP group. ***p < 0.005 and ###*p* < 0.005. The asterisk (*) represents statistically significant difference between the indicated group and the Con group.

To confirm the cell type to which Reduning applied, IF was used to investigate the expression and localization of p-ERBB2 in each group. As shown in 7A, p-ERBB2 was co-located with F4/80. As shown in Figure 7B, compared with the sham group, p-ERBB2 was significantly enhanced in the CLP group (*p* < 0.01). In comparison with the CLP group, p-ERBB2 was sharply reduced after RDN intervention (*p* < 0.01).

### 3.7 The ERBB2-p38 MAPK signaling pathway activated in AMs of pulmonary fibrosis tissue

**FIGURE 8:**
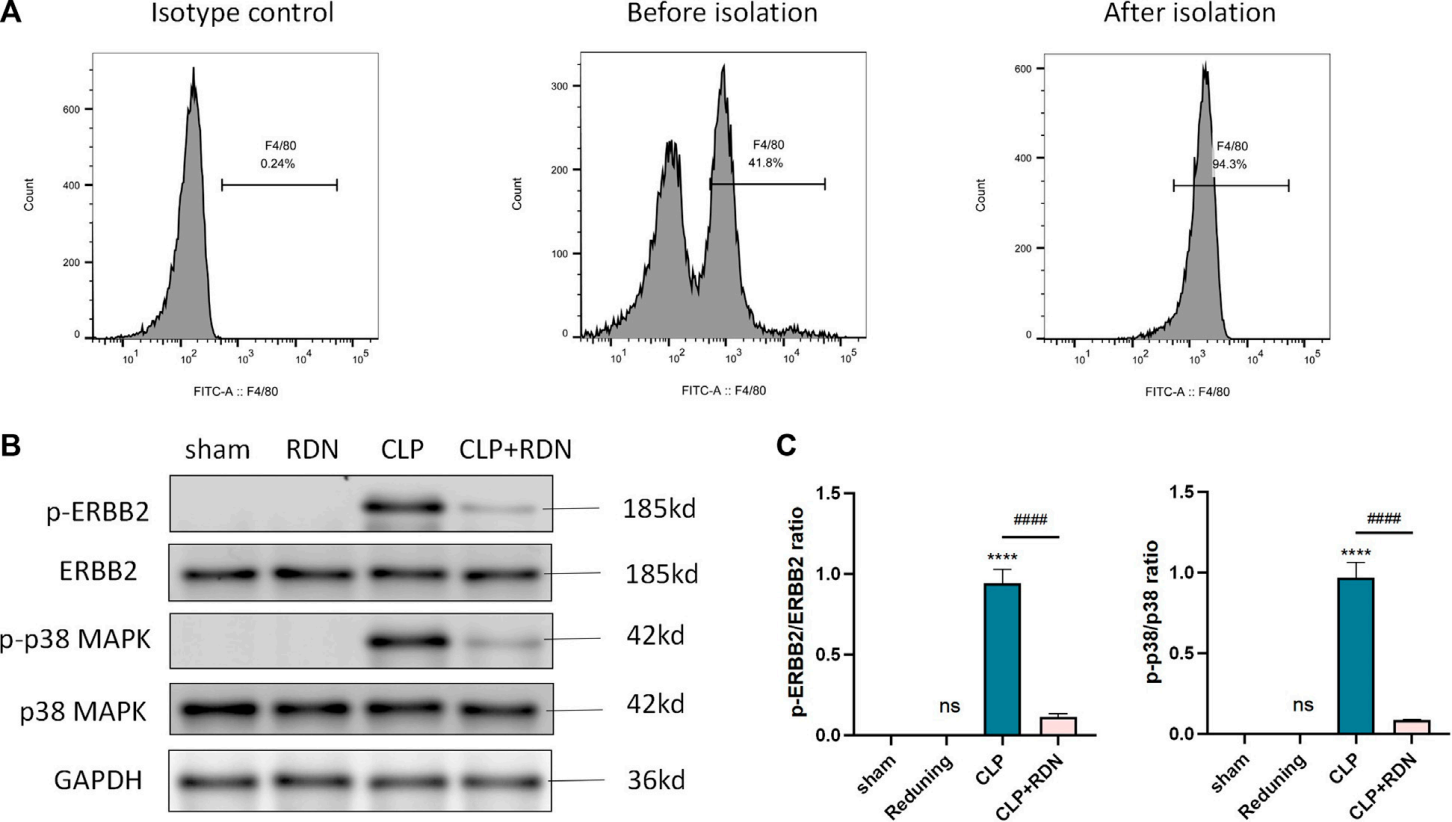
Expression of the ERBB2-p38 MAPK pathway in AMs. **(A)** Flow cytometry was used to isolate AMs. **(B–D)** The expression levels of the ERBB2-p38 MAPK pathway-related proteins were detected by Western blot. Data are presented as mean ± SD (*n* = 3 per group) of the representative data from three independent experiments; ****p < 0.001 and ####*p* < 0.001. The asterisk (*) represents statistically significant difference between the indicated group and the Con group.

To further detect the underling mechanism of RDN, AMs were isolated from the lung tissue. We detected the ERBB2-p38 MAPK signaling pathway-related proteins in each group. As illustrated in Figures 8B,C, the expression levels of p-ERBB2/ERBB2 and p-p38/p38 MAPK which were enhanced in the CLP group were significantly dampened by Reduning in the CLP+Reduning group (*p* < 0.001). There is no significant difference between the Reduning group and the sham group (*p* > 0.05).

### 3.8 RDN could reduce the M2 polarization of AMs in CLP-induced pulmonary fibrosis

**FIGURE 9:**
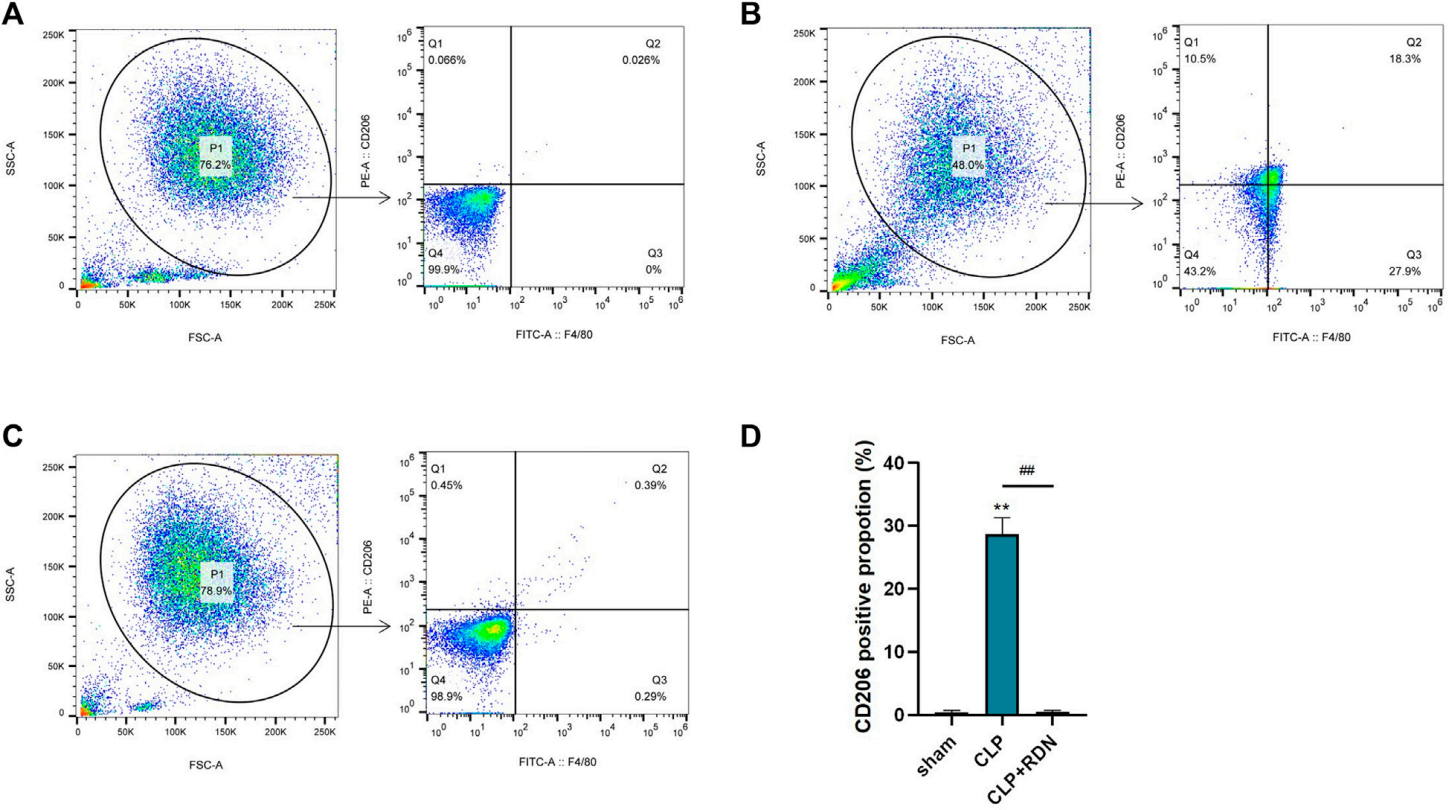
The impact of Reduning on the polarization of AMs. The expression of CD206 on alveolar macrophages in the sham group **(A)**, CLP group **(B)**, and CLP+100 mg/kg RDN group **(C)** was detected by flow cytometry and statistically plotted **(D)**. Data are presented as mean ± SD (n = 3 per group) of the representative data from three independent experiments; ****p < 0.001 and ####*p* < 0.001. The asterisk (*) represents statistically significant difference between the indicated group and the Con group.

To clarify the impact of RDN on the polarization of AMs, flow cytometry was used to measure the expression of CD206. As shown in Figure 9, compared with the sham group, CD206 in the CLP group was evidently promoted. Reduning could alleviate the expression level of CD206. Also, the cell apoptosis proportions were 0.47 ± 0.16, 28.63 ± 1.53, and 0.51 ± 0.17 for the sham group, the CLP group, and the CLP+Reduning group, respectively (*p* < 0.001).

## 4 Discussion

ARDS is a common clinically acute critical illness with an average mortality of 30–40% (Meyer et al., 2021). Sepsis is the most common cause of ARDS, accounting for about half of all cases. Pulmonary fibrosis is a late complication of ALI/ARDS, marked by fibroblast proliferation and excessive deposition of extracellular matrix (Matthay et al., 2019). Pulmonary fibroproliferative changes in ARDS patients have been proved to be a key factor for poor prognosis and high mortality (Raghu et al., 2011; Burnham et al., 2014). Studies have pointed out that sepsis-induced pulmonary fibrosis is related to the proliferation and invasion of fibroblasts, chronic inflammatory reaction of pulmonary endothelial cells, and polarization of alveolar macrophages (Keshari et al., 2014; Kishore and Petrek, 2021). COVID-19 has broken out in succession around the world, among which severe patients eventually develop ARDS (Bonaventura et al., 2021). Studies have confirmed that SARS-CoV-2 triggers the proliferation of ARDS (Wendisch et al., 2021). However, specific therapies to reduce sepsis-induced pulmonary fibrosis are still lacking in current studies.

Reduning has pharmacological effects such as clearing heat, dispersing wind, and being antiviral and antibacterial, and has been mainly used to treat infectious emergencies in clinical practice. In recent years, Reduning and its active components have been widely studied in the prevention and treatment of pulmonary fibrosis. For example, it was identified that beta-sitosterol could significantly suppress epithelial–mesenchymal transition (EMT) by the governing TGF-β1/Snail pathway, thus downregulating the TGF-β1-induced fibrotic proteins in human alveolar epithelial cells (Park et al., 2019). In addition, kaempferol significantly inhibited pulmonary inflammation and 28-day silicosis-induced pulmonary fibrosis in a 7-day silicosis model, and this effect was related to the enhancement of autophagy (Liu et al., 2019). Moreover, quercetin is reported to reverse the resistance to death ligand-induced apoptosis in mice model by promoting Fas Ligand receptor and caveolin-1 expression and inhibiting AKT activation, alleviating the progression of bleomycin-induced pulmonary fibrosis (Hohmann et al., 2019).

In this experiment, we explored the possible targets of Reduning in the prevention of sepsis-induced pulmonary fibrosis using network pharmacological methods. We identified key active components, stigmasterol, kaempferol, quercetin, beta-sitosterol, and corymbosin, and key targets, SRC, TNF, CASP3, GAPDH, and ERBB2, by PPI. We performed molecular docking between key targets and key active components and selected the top five according to the absolute value of binding energy for molecular docking display. As can be seen in the table, the binding energies of stigmasterol, quercetin, and beta-sitosterol complexes with ERBB2 were in the top five, indicating that many active components of Reduning had good binding activities with ERBB2. Molecular docking and MDs can explain the mechanism of interactions between molecules in depth, and provide an important tool for predicting the binding types and interaction modes of biomacromolecule complexes. Currently, they have become an important research method for explaining biological mechanisms (Hollingsworth and Dror, 2018). MDs were to observe the stability and kinetic characteristics of the combination. Stigmasterol, quercetin, and beta-sitosterol were found to be stably bound to ERBB2. ERBB2, located on chromosome 17q21, is a proto-oncogene encoding human epidermal growth factor receptor-2 (HER2) protein. The HER2 protein is a transmembrane protein with tyrosine protein kinase activity, belonging to the epidermal growth factor receptor family. It is mostly used as an independent prognostic indicator of breast cancer, is also an important target in tumor targeted therapy, and plays an important role in the regulation of the immune microenvironment (Smith et al., 2021; Tolmachev et al., 2021). In our previous study on the possibility of Reduning in the treatment of monkeypox, we also found that ERBB2 might be the target of Reduning in the treatment of monkeypox (Wang et al., 2022b). Recently, scholars have gradually paid attention to the relationship between ERBB2 and fibrosis, and they found that inhibition of HER2 can improve myocardial fibrosis caused by myocardial infarction or diabetes by regulating the TGF-β pathway (Amani et al., 2020; Humeres et al., 2021). ERBB2 expression was found to be increased in both pulmonary fibrosis patients and animal models, and the activation of phosphorylated ERBB2 in normal lung fibroblasts may be the cause of fibroblast invasion and pulmonary fibrosis, while antagonizing HER2 can weaken the invasion ability of fibroblasts and improve pulmonary fibrosis (Liu et al., 2022; Li et al., 2011).

GO and KEGG enrichment analysis showed that the shared targets of diseases and drugs were mostly enriched in the MAPK cascade signaling pathway, protein phosphorylation, and kinase activation pathway. The ERBB2–MAPK pathway has been extensively studied in cancer. P38γ MAPK is downstream of ERBB2 and plays an important role in alcohol-enhanced aggressiveness of breast cancer (Xu et al., 2016). Prolactin in breast cancer cells could induce the phosphorylation of ERBB2/HER2, which in turn activates the downstream RAS/MEK/ERK pathway, leading to the proliferation of breast cancer (Kavarthapu et al., 2021). Fan et al. showed that the ERBB2 S310F mutation could promote auto-phosphorylation to activate the downstream MAPK pathway and further promote the growth of gallbladder cancer (Fan et al., 2022). As the key compounds of Reduning, stigmasterol, quercetin, and beta-sitosterol have the ability to reduce protein phosphorylation (Sook et al., 2014; Seo et al., 2016; Sampath et al., 2021). Quercetin was confirmed to reverse tamoxifen resistance in breast cancer cells with downregulation of HER2 (Wang et al., 2015). Since both cancer and fibrosis have been reported to be closely associated with chronic inflammation (Katoh, 2018), we hypothesized that the active components stigmasterol, quercetin, and beta-sitosterol of Reduning may inhibit p-ERBB2/HER2-MAPK to block the progression of sepsis-induced pulmonary fibrosis.

Then, *in vivo* and *in vitro* experiments were performed to verify the assumption. CLP operation was applied to mimic a sepsis-induced pulmonary fibrosis mice model. Masson staining and IHC showed that Reduning could attenuate CLP-induced fiber deposition *in vivo*. Next, we explored the cell types in which RDN improved the progression of pulmonary fibrosis by downregulating the activation of ERBB2. As illustrated in the IF results, p-ERBB2 is co-localized with AMs. M1 macrophages are pro-inflammatory cells that have bactericidal and tumor-killing activities, while M2 macrophages are involved in tissue remodeling and promoting fibrosis and mainly secrete anti-inflammatory factors such as TGF-β1 and IL-10. Studies have shown that pulmonary fibrosis is directly related to the abnormal polarization of AMs (Pan et al., 2020). In co-culture models of macrophages and alveolar epithelial cells/fibroblasts, macrophages have been found to promote epithelial–mesenchymal transition (EMT) and fibroblast migration of alveolar epithelial cells (Li et al., 2019). For example, S100A4 released by macrophages promotes pulmonary fibrosis by activating Sphingosine-1-phosphate (S1P)-related lung fibroblasts (Li et al., 2018). Abnormal macrophage function promotes fibrosis and is related to the MAPK pathway. Goda found that the specific deletion of transcription factor FOXM1 in mouse macrophages (myFoxm1−/−) could exacerbate PF by increasing p38 MAPK signaling in macrophages (Goda et al., 2020). Therefore, we further detected the ERBB2-p38 MAPK signaling pathway in AMs isolated from the indicated mice groups. Results showed the activation of ERBB2 and p38 in the CLP group could be alleviated by Reduning. Flow cytometry has also indicated that AMs were polarized toward the M2 type in the CLP group but reduced after Reduning intervention. Therefore, the results partly elucidated that Reduning may attenuate sepsis-induced pulmonary fibrosis by regulating the ERBB2/HER2-p38 MAPK signaling pathway in AMs.

## 5 Limitation

This study innovatively proposed that Reduning could prevent sepsis-induced pulmonary fibrosis through ERBB2 as a target. Although *in vivo* and *in vitro* experiments were used to verify the results of network pharmacology, this study still fell short of unfolding the underlying mechanism between AMs and fibroblasts. We could further explore it in the future.

## 6 Conclusion

In conclusion, downregulating the ERBB2-p38 MAPK signaling pathway in AMs may prevent sepsis-induced pulmonary fibrosis, pointing to a potential therapeutic target. In this study, molecular docking and molecular dynamics simulation were used for the first time to explore the possibility of Reduning in the prevention of sepsis-induced pulmonary fibrosis. Network pharmacology and molecular docking results showed Reduning may regulate ERBB2 phosphorylation and the MAPK signaling pathway through stigmasterol, quercetin, and beta-sitosterol, thereby preventing sepsis-induced pulmonary fibrosis. The reliability of the aforementioned results was verified by *in vivo* and *in vitro* experiments, and the kinetic characteristics of the complexes were explained. This study provides a new clue to the molecular mechanism of Reduning in the prevention of sepsis-induced pulmonary fibrosis and also provides a basis for its clinical application.

## Data Availability

The datasets presented in this study can be found in online repositories. The names of the repository/repositories and accession number(s) can be found in the article/supplementary material.
